# Decision-making in everyday moral conflict situations: Development and validation of a new measure

**DOI:** 10.1371/journal.pone.0214747

**Published:** 2019-04-01

**Authors:** Nina Singer, Ludwig Kreuzpointner, Monika Sommer, Stefan Wüst, Brigitte M. Kudielka

**Affiliations:** 1 Department of Medical Psychology, Psychological Diagnostics and Research Methodology, University of Regensburg, Regensburg, Germany; 2 Department of Psychiatry and Psychotherapy, University of Regensburg, Regensburg, Germany; 3 Department of Psychology, Ludwig-Maximilians-University of Munich, Munich, Germany; 4 Institute of Experimental Psychology, University of Regensburg, Regensburg, Germany; Middlesex University, UNITED KINGDOM

## Abstract

In everyday life, we are often confronted with morally conflicting social interaction situations. Therefore, the main objective of the present set of studies was the development and validation of a new measure to assess decision-making in everyday moral conflict situations. All vignettes required a decision between an altruistic versus an egoistic behavioral response alternative. In three independent surveys (*N* = 200), we developed a 40-items measure with preferable mean rates of altruistic decisions (Study 1), clear representation of altruistic and egoistic response classes (Study 2), unambiguousness of social closeness classifications (socially close vs. socially distant protagonists; Studies 1 and 2), and high similarity to reality ratings (Studies 1 and 2). Additionally, we developed two parallelized item sets for future use in within-subjects design studies and investigated the measurement properties of our new scale (Studies 1 and 3). Results of Rasch model analyses and classical test theory fit indices showed unidimensionality and confirmed the appropriateness of the fragmentation into two parallelized item sets. Notably, in our data, there were neither effects of social closeness nor gender on the percentage of altruistic decisions. In sum, we propose the Everyday Moral Conflict Situations (EMCS) Scale as a promising new measurement tool that may facilitate further research in different research areas due to its broad applicability.

## Introduction

For humans and most animals, the social interaction with conspecifics is a common everyday life activity [1]. Thereby, we humans are often confronted with morally conflicting social interaction situations. According to Christensen and colleagues [2], moral conflicts are situations in which someone is pulled in contrary directions by rival moral reasons. They can, amongst others, occur when deciding between a personal interest versus an accepted moral value.

In experimental studies, decision-making in moral conflict situations is typically investigated by moral dilemmas presented as short stories about situations involving moral conflicts (see [2] for review). To date, most studies in various research areas (e.g., judgment and decision-making, cognitive neuroscience, social psychology, or stress research) have applied abstract moral dilemmas. The trolley problem, for instance, prompts the participant to decide whether or not one person should be actively sacrificed in order to save a greater number of people (e.g., [3–7]). Such abstract moral dilemmas with utilitarian versus deontological response alternatives were once inspired by thought experiments of famous historical moral philosophers like Immanuel Kant (1724–1804) and experimental studies of developmental psychologists like Jean Piaget (1896–1980) or Lawrence Kohlberg (1927–1987). Over the last decades, abstract moral dilemma research has provided us many important and fascinating insights into the processes involved in moral judgment (e.g., regarding the interplay between cognition and emotion; [3, 4, 8, 9]) using very different paradigms and scales (see for example [10] for a recently developed abstract moral decision-making scale, which has been successfully applied by [9]). Nevertheless, methodological concerns about the interpretation of the results of abstract moral dilemma research have been raised in the last years (e.g., [11, 12]). Additionally, despite of recent attempts to transfer trolley-style moral dilemmas to real life situations (e.g., [13]), such sacrificial dilemmas lack external and ecological validity [14, 15] and can only hardly be transferred to situations experienced in everyday life [16].

Thus, current moral dilemma research comprises no longer only abstract reasoning about moral dead-or-life situations (e.g., [3, 4]) but also research on moral decision-making in a variety of daily life situations (e.g., [17, 18]). Applying ecological momentary assessment in a large study sample (*N* = 1.252 participants), Hofmann et al. [18] repeatedly assessed moral or immoral acts and experiences in everyday life. The authors were able to confirm five areas of human morality (care/harm, fairness/unfairness, loyalty/disloyalty, authority/subversion, and sanctity/degradation) as originally proposed by the Moral Foundations Theory (MFT; [19, 20]). Moreover, honesty/dishonesty was the third most frequently mentioned dimension regarding morality in everyday life and, thus, emerged as another important category [18, 21].

With regard to research in laboratory settings, several scholars (e.g., [16, 22, 23]) recently developed everyday moral dilemmas. Everyday moral dilemmas are short vignettes describing hypothetical everyday life situations. The vignettes require decisions between the fulfilment of a moral standard or social obligation towards another person versus a personal-oriented hedonistic behavior that would explicitly not cause serious bodily harm or legal consequences [16]. The given response alternatives are typically altruistic (e.g., helping an old woman who is in distress) versus egoistic (e.g., catching the waiting bus home; see [23] or [24] for further examples).

However, to date, there is a paucity of validated everyday moral decision-making paradigms. To the best of our knowledge, only Starcke et al. [23] and Rosen et al. [22] reported validation studies of their everyday moral conflict scenarios as supplementary material to their manuscripts. Both Starcke et al. [23] and Rosen et al. [22] subdivided their items into high- versus low-emotional dilemmas, but many of their high-emotional dilemmas do not describe common everyday life situations (e.g., deciding about leaving your partner who is suicidal), which is a potential constraint of these paradigms. By contrast, the scenarios developed by Sommer and colleagues [16] do rather reflect very typical daily life situations and proved useful in several experimental studies [16, 24, 25]. However, a more comprehensive validation of such everyday moral conflict situations would be highly desirable. Furthermore, all of the above-mentioned vignettes were only developed for use in between-subject design studies. Thus, to date, no parallelized item sets of everyday moral decision-making paradigms for use in within-subjects design studies are available (e.g., to be applied before and after an experimental manipulation like acute stress induction). Such material would enable researchers to control for potential effects due to interindividual variability in trait variables (e.g., personality traits; see [26]) and, thus, help to achieve more statistical power [27]. Moreover, the availability of parallelized item sets would be of high advantage as simple retesting with identical stimulus material is not advisable because memory effects could confound the findings.

Therefore, the main objective of the present studies was the development and validation of a new measure with two parallelized item sets assessing decision-making in everyday moral conflict situations. We operationalized everyday moral decision-making as the readiness to act altruistically, defined as the degree of following accepted moral values instead of fulfilling personal interests (see [16]). Based on the MFT [19–21] and the results of a recent ecological momentary assessment study [18], we developed the Everyday Moral Conflict Situations (EMCS) Scale, a new set of everyday moral dilemmas with the dimensions of care/harm, fairness/unfairness, loyalty/disloyalty, and honesty/dishonesty. We decided against including items referring to authority/subversion in order to avoid potential confounding influences of legal consequences [16]. Moreover, the dimension of sanctity/degradation was considered as inappropriate for our new scale because in our view, religious standards cannot be clearly assigned to altruistic versus egoistic behavior.

In addition to scale development and validation, we aimed at investigating if everyday moral decision-making depends on the social closeness of the protagonists. As revealed in a recent review, the relatedness of the participant to the story characters is an important experimental design parameter in moral dilemma research [2]. Therefore, social closeness could be one further potential modulating factor of everyday moral decision-making (apart from the emotionality of a situation, which has already been examined in previous studies; see [22, 23]). Consistently, several studies have shown that both abstract and everyday moral decision-making differ depending on the closeness of the relationship with the target person (e.g., [5, 17, 28–31]). With regard to everyday moral decision-making in laboratory settings, so far only Zhan and colleagues [31] investigated the impact of the social closeness of the protagonists. They observed that participants made less altruistic decisions, took more time for their decisions, and rated the situations as emotionally more negative if the moral conflicts involved strangers versus friends and acquaintances. Presuming that altruistic decisions are some kind of generous acts, this finding is also in line with studies in social psychology on social discounting, showing that generosity decreases hyperbolically with increasing social distance between the donor and the recipient [32–37]. Thus, we experimentally varied the social closeness of the protagonists in our scenarios (socially close vs. socially distant) and hypothesized a lower percentage of altruistic decisions for scenarios involving socially distant protagonists as compared to stories involving socially close target persons.

Furthermore, several studies reported gender differences in abstract moral decision-making (e.g., [7, 38–41]). Historically, this line of research can be traced back to Gilligan [42], who proposed that males differ from females in their approach to moral reasoning (justice focused vs. care focused; see [43]). Moreover, in related research disciplines, it has been demonstrated that females are more honest [44, 45] and also more altruistic than males regarding generosity in the Dictator Game (see [46] and [47] for two recent meta-analyses). We therefore hypothesized gender differences in everyday moral decision-making as well. Thus, we equally recruited males and females for participation in our surveys and counterbalanced our new vignettes in terms of the gender of the protagonists.

In sum, we conducted three independent validation surveys in order to (a) develop and evaluate the psychometric properties of our new 40-items EMCS Scale (20 items with socially close and 20 items with socially distant protagonists; Studies 1 and 2), (b) develop two parallelized item sets for future use in within-subjects design studies (Studies 1 and 3), and (c) evaluate the measurement properties of our new scale and its different subsets (Studies 1 and 3). In addition to scale development and validation, we examined two research questions regarding the impact of the social closeness of the protagonists as well as potential gender differences in everyday moral decision-making. As a supplementary analysis, we finally examined if the answers to items with socially close protagonists differed depending on the actual existence of the target persons (e.g., partner, brother, sister) in the real lives of the participants. The rationale behind this supplementary investigation was to rule out the possibility that the actual (non-)existence of the respective target persons in the lives of participants might be a confounding factor that influences everyday moral decision-making. It should be of note that this exploratory question had no direct empirical foundation yet.

## Materials and methods

### Participants

Altogether, *N* = 200 volunteers (100 males, 100 females, mainly students) aged 18–43 years (mean age ± *SD* = 22.55 ± 4.22 years), participated in three separate surveys, which took place at the University of Regensburg. The study protocols were approved by the local ethics committee of the University of Regensburg, Germany and performed in line with the Declaration of Helsinki. All participants gave written informed consent to their participation in the surveys and received a monetary compensation of 4 €, chocolate, or course credit, respectively.

### Everyday moral conflict situations

Initially, a pool of 60 hypothetical everyday moral conflict situations with two forced-choice response alternatives (altruistic vs. egoistic) was created. For that purpose, we modified the 28 everyday moral dilemmas originally developed by Sommer et al. [16], and developed 32 additional new scenarios with the contents of care/harm, fairness/unfairness, loyalty/disloyalty, and honesty/dishonesty [18, 19]. We aimed at creating only everyday moral conflict situations that could easily occur in daily life in order to achieve high external and ecological validity of our new measure.

All stories were constructed in first-person narrative and followed a three-sentence structure. We systematically varied the social closeness of the protagonists (two categories: socially close vs. socially distant), so that in half of the stories, the protagonist was a socially close person (e.g., father, partner, friend), and in the other half he or she was socially distant (e.g., cashier, barkeeper, stranger). Moreover, the conflict situations were counterbalanced in terms of the gender of the target persons (for examples see Table 1; the complete final item set is provided in S1 Table). Using Research Randomizer [48], a fixed random presentation order of the 60 (Studies 1 and 2) or 40 (Study 3) scenarios was created before application. This order was then realized for all participants.

**Table 1:** Item examples of the EMCS scale. Examples of the everyday moral conflict situations with corresponding response alternatives (altruistic vs. egoistic) subdivided into scenarios with socially close and socially distant protagonists.

Everyday moral conflict situation	Response alternatives (altruistic vs. egoistic)
***Examples of scenarios with socially close protagonists***	
It is the soccer world cup and the final match is on TV. I am a big soccer fan and very excited about the game. All of a sudden, a friend of mine who is not feeling well gives me a call and wants to meet up with me right now. What do I do?	I meet up with my friend. I watch the soccer game.
I have promised my partner to go to the company party with him/her. He/she has already signed both of us up. Now I realize that I would urgently need the time to prepare for an important exam. What do I do?	I keep my promise. I prepare for the exam.
I am at the airport, ready to leave on a long-planned holiday. While I am standing at the check-in counter, my mother gives me a call. She tells me that my father had a little accident and was admitted to the hospital. What do I do?	I cancel the holiday. I take the flight anyway.
***Examples of scenarios with socially distant protagonists***	
I want to sell my old car. I know that the car’s radiator actually needs to be exchanged urgently. A man who does not notice the problem with the radiator offers to pay a good price in cash right away. What do I do?	I mention the defect. I keep quiet about the defect.
I am at the checkout of a supermarket and I want to pay for my groceries, which cost 8 €. I give a 10 € bill to the cashier. She accidentally gives me back 4 € instead of 2 €. What do I do?	I return the money. I keep the money.
I am running to catch a bus that is about to leave and that only runs once every hour. In front of me, several items drop out of the purse of a woman with two small children. Except for me, there is no one else around to help the woman. What do I do?	I help the woman. I run to the bus.

The order of the two corresponding response alternatives was counterbalanced in our surveys.

In all our surveys, the items were originally presented in German language. However, for the purpose of presentation in the manuscript, the items were in a first step translated into English language by four independent persons (including NS and BMK). In a second step, a consentaneous version was reached by discussion. In a third step, the obtained English translation was thoroughly reviewed by an English native speaker for colloquial speech and concordance with the German original (AP; see Acknowledgments).

### Procedure

#### General procedure

Depending on study assignment, participants had to fill out one of four different paper-pencil questionnaires, which all took about 15–30 minutes to complete. The questionnaires consisted of 60 (Studies 1 and 2) or 40 (Study 3) everyday moral conflict situations. Before responding to the everyday moral conflict situations, all participants provided demographic information (age, gender, occupation). Prior to the tasks, participants were given a standardized written instruction that reminded them to put themselves into the positions of the protagonists described in the conflict stories and to answer all questions spontaneously.

#### Study 1

In Study 1, the complete set of 60 stories was given to *n* = 50 participants (25 males, 25 females; aged 23.38 ± 4.47 years). After every conflict situation, the question “What do I do?” as well as the altruistic and the egoistic response alternatives were presented in a counterbalanced order. For every item, participants first had to indicate their decision. Subsequently, they were asked to rate the social closeness of the protagonist on a 7-point Likert scale ranging from 1 = *very socially distant* to 7 = *very socially close* and the similarity to reality of the presented situation on a 7-point Likert scale ranging from 1 = *very far from reality* to 7 = *very close to reality*. For the purpose of analysis, altruistic decisions were coded as “1” and egoistic decisions as “0”; then the percentage of altruistic decisions was calculated. The ratings of social closeness and similarity to reality were each aggregated to calculate their arithmetic means.

In order to examine if the answers to the items with socially close protagonists differed depending on the actual existence of the target persons in the real lives of the participants, subjects additionally had to indicate at the end of the survey if they currently were in a partnership, and if they had a father, mother, brother, sister, uncle, aunt, grandfather, or grandmother who they knew or had known in the past.

#### Study 2

In Study 2, *n* = 50 participants (25 males, 25 females; aged 24.08 ± 4.44 years) were confronted with the same 60 everyday moral conflict situations as in Study 1. However, differing from Study 1, where we assessed the item difficulties of the everyday moral conflict scenarios via binary decisions between given altruistic versus egoistic responses, the main aim of Study 2 was to validate the *a priori* defined altruistic and egoistic response alternatives of the stories. Therefore, in Study 2, we replaced the answer options at the end of the stories in two different ways. First, every story was finished by a statement that indicated altruistic behavior of the acting person (e.g., “My decision: I help the woman”) and second, each story was ended by a description of the actor behaving egoistically (e.g., “My decision: I take the bus”). We then created two different versions of the survey, each with 30 given altruistic and 30 given egoistic responses, and randomly assigned *n* = 25 participants (12 males, 13 females) to one version of the questionnaire (Study 2A) and *n* = 25 participants (13 males, 12 females) to the other version (Study 2B). In both subversions, we asked the participants to judge the given responses on a 7-point Likert scale ranging from 1 = *egoistic* to 7 = *altruistic*. Altruistic and egoistic response options were presented in a counterbalanced order. Similar to Study 1, participants subsequently had to rate the social closeness of the protagonists on a 7-point Likert scale ranging from 1 = *very socially distant* to 7 = *very socially close* as well as the similarity to reality of the presented situations on a 7-point Likert scale ranging from 1 = *very far from reality* to 7 = *very close to reality*. Furthermore, at the end of the survey, participants also had to indicate if they currently were in a partnership, and if they had a father, mother, brother, sister, uncle, aunt, grandfather, or grandmother who they knew or had known in the past.

For the purpose of analysis, the ratings of the altruistic and egoistic responses, social closeness, and similarity to reality were each aggregated to calculate their arithmetic means. As the ratings for social closeness and similarity to reality were part of the Studies 1 and 2, we merged these data resulting in a total sample of *n* = 100 participants (50 males, 50 females; aged 23.73 ± 4.45 years) for respective analyses.

#### Study 3

In Study 3, *n* = 100 participants (50 males, 50 females; aged 21.36 ± 3.64 years) were confronted with 40 everyday moral conflict situations (20 items with socially close and 20 items with socially distant protagonists), which were selected based on the results of Studies 1 and 2. Since one aim of Study 3 was the development of two parallelized item sets (set A and B), which could then be used in repeated-measurement design studies, participants were only confronted with the question “What do I do?” and the two possible response alternatives (altruistic vs. egoistic) in the fixed random order.

For the purpose of analysis, altruistic decisions were coded as “1” and egoistic decisions as “0”; then the percentage of altruistic decisions was calculated. As the type of decision (altruistic vs. egoistic) was assessed in Studies 1 and 3, we also merged the data of these surveys. Thus, we developed the two parallelized item sets for future use in within-subjects design studies and assessed the test and measurement properties of our new scale based on a total sample of *n* = 150 participants (75 males, 75 females; aged 22.04 ± 4.04 years).

### Statistical analyses

Statistical analyses were performed using the IBM SPSS (version 25.0) statistical software package and R (version 3.5.1; [49]) with the packages psych [50], eRm [51], foreign [52], and psychometric [53]. The significance level was set at α = .05; all testing was two-tailed. Results in the text are given as mean ± standard deviation (*M* ± *SD*). For all between- and within-group comparisons, Cohen's *d* is reported as a measure of effect size.

The results section is structured into two main parts. In the first main part, we present analyses concerning the development and validation of our new 40-items EMCS Scale as well as the development of two parallelized 20-item sets (each ten scenarios with socially close and socially distant protagonists) for future use in within-subjects design studies. For the two parallelized sets A and B, we used Wilks L_mvc_ tests to demonstrate parallelism [54]. The procedure by Wilks tests the hypothesis that the means are equal, the variances are equal, and the covariances are equal. The test statistic is based on the weighted differences of the subsample means with the grand mean and the ratio of subsample and complete sample (co)variances, which are shown to be chi^2^-distributed when the data meets hypothesis (see [54], formula 1.4). Moreover, we report the measurement qualities based on Rasch model analyses and classical test theory fit indices of the complete 40-items EMCS Scale and its two subsets A and B.

The second main part presents first content-related analyses regarding our newly developed EMCS Scale. We used Welch-tests [55] to examine the effects of the social closeness of the protagonists and the gender of our participants on everyday moral decision-making. Furthermore, we explored possible effects of the actual existence of a socially close protagonist in the lives of the participants on response tendencies in everyday moral conflict situations. We also ran Welch-tests to analyze between-group mean differences in the percentage of altruistic decisions, social closeness ratings, and similarity to reality ratings.

## Results

### Development and scale characteristics of the EMCS Scale

#### Scale development

The new EMCS Scale was developed in a six-step procedure. Overall, we selected 40 out of initially 60 items (20 items with socially close and 20 items with socially distant protagonists) based on four criteria: (1) preferable mean rate of altruistic decisions, (2) clear representation of altruistic and egoistic response classes, (3) unambiguousness of social closeness classifications, and (4) high similarity to reality ratings.

In a first step, we calculated the item difficulties of all 60 items. This analysis was based on the data of Study 1. In order to avoid ceiling or floor effects as much as possible, we excluded 16 items (ten items with socially close and six items with socially distant target persons) due to a relatively high (≥ 0.8) or low (≤ 0.2) percentage of altruistic decisions.

In a second step, we validated the *a priori* defined behavioral response classifications. We therefore analyzed if altruistic decisions were clearly perceived as altruistic and egoistic decisions as egoistic. This analysis was based on the data of Study 2. All of the 44 remaining items provided acceptable mean altruistic and egoistic response ratings (i.e., for altruistic responses above and for egoistic responses below the average scale value of 4).

In a third step, we checked the *a priori* defined social closeness classifications, that is, if socially close protagonists were clearly perceived as socially close and socially distant target persons as socially distant. This analysis was based on the data of Studies 1 and 2. All items showed social closeness ratings in the expected direction: the means of items with socially close protagonists were above the average scale value of 4, and the means of items with socially distant protagonists below 4.

In a fourth step, we checked the similarity of the stories to everyday life situations. This analysis was based on the data of Studies 1 and 2. One item with a socially distant protagonist showed a somewhat lowered mean similarity to reality rating of 3.43 and was therefore excluded. The similarity to reality ratings of the remaining items reached at least a mean rating above 3.5 (range: 3.57 to 5.74).

In a fifth step, we excluded three further items with socially distant protagonists that showed the least favorable combination of the four aforementioned criteria in order to reach a final 40-items set with 20 socially close and 20 socially distant protagonists.

In a sixth step, the final 40 items were adjusted in terms of the gender of the protagonists. For that purpose, the gender of eight target persons (five socially close and three socially distant) was changed to achieve an equal gender distribution.

#### Scale characteristics

Regarding our final 40-items EMCS Scale, participants chose the altruistic response alternative in an average of 59.70% (± 14.59%, with 0.40% missings) of the everyday moral conflict situations, which was significantly higher than 50% (*t*(149) = 8.15, *p* < 0.001). The item statistics for all final 40 items of the EMCS Scale on single item basis (subdivided into items with socially close and socially distant protagonists) can be found in S1 and S2 Tables.

### Development of two parallelized item sets for use in within-subjects design studies

In order to develop two parallelized item sets for future use in within-subjects design studies, we took the complete 40-items EMCS Scale and generated item pairs based on social closeness, gender of the protagonists, and story content in a first step. In a second step, the 20 item pairs were split into two different item sets (each ten scenarios with socially close and socially distant protagonists). In a third step, the allocation of the items was pairwise interchanged until we achieved two parallelized item sets regarding item difficulty (percentage of altruistic decisions) and their properties (social closeness ratings and similarity to reality ratings).

In the final version, the items 2, 5, 7, 8, 9, 10, 11, 12, 16, 20 (socially close protagonists) and 24, 26, 27, 30, 31, 35, 37, 38, 39, 40 (socially distant protagonists) were assigned to set A; the items 1, 3, 4, 6, 13, 14, 15, 17, 18, 19 (socially close protagonists) and 21, 22, 23, 25, 28, 29, 32, 33, 34, 36 (socially distant protagonists) became part of set B (see last column in S1 Table).

Regarding the percentage of altruistic decisions, the comparison of the means and standard deviations of the sets A and B showed a very close to one value of L_mvc_ = 0.997 (*p* = 0.78), indicating that there were no significant differences between set A (59.38% ± 16.47) and set B (60.03% ± 15.95). The Spearman-Brown corrected split-half reliability was 0.77. Moreover, the same results arose for the comparison of the sets A and B including only the items with socially close protagonists (set A 60.30% ± 18.21, set B 60.83% ± 17.79; L_mvc_ = 0.999, *p* = 0.90) or the items with socially distant protagonists (set A 58.51% ± 20.66, set B 59.23% ± 20.98; L_mvc_ = 0.998, *p* = 0.88), respectively. The mean social closeness ratings for the items with socially close protagonists (set A 5.84 ± 0.49, set B 5.85 ± 0.52; *d* = -0.002) and for the items with socially distant protagonists (set A 1.76 ± 0.80, set B 1.78 ± 0.75; *d* = -0.03) also hardly differed between the two constructed item sets. Moreover, there was no relevant difference between the mean similarity to reality ratings in set A (4.67 ± 0.89) and set B (4.66 ± 0.85; *d* = 0.004).

### Test and measurement properties of the EMCS Scale and its two item sets A and B

We tested the measurement qualities of our new scale by fitting a one-parameter logistic Rasch model (1 PL IRT; e.g., [56, 57]) for the complete EMCS Scale and its two subsets A and B. The model fit was tested with the Andersen LR-test [58] based on a median split and a graphical model check. We used the defaults of the eRm packages reducing the R code to: eRm::LRtest(eRm::(data[,items])). For testing the unidimensionality of the EMCS Scale and the appropriateness of the fragmentation into the two item sets A and B, we used the Martin-Loef LR-test [59]. With the Martin-Loef LR-test, the likelihood of the unidimensionality assumption is compared with the likelihood of two or more (in this study up to four) dimensions. A unidimensional interpretation of the measure is more appropriate, the lesser the enhancement of a more specific structure (multiple dimensions; R code: eRm::MLoef(eRM::RM(data[,items])). Thereby, the complete 40-items set was in a first step split into two parts based on a median split regarding the items’ raw scores. In a second step, we checked the fit for dividing the items into the sets A and B. Moreover, we split the items into four parts depending on their belonging to set A or B and the social closeness of the protagonists (set A socially close, set A socially distant, set B socially close, set B socially distant).

**Fig 1:**
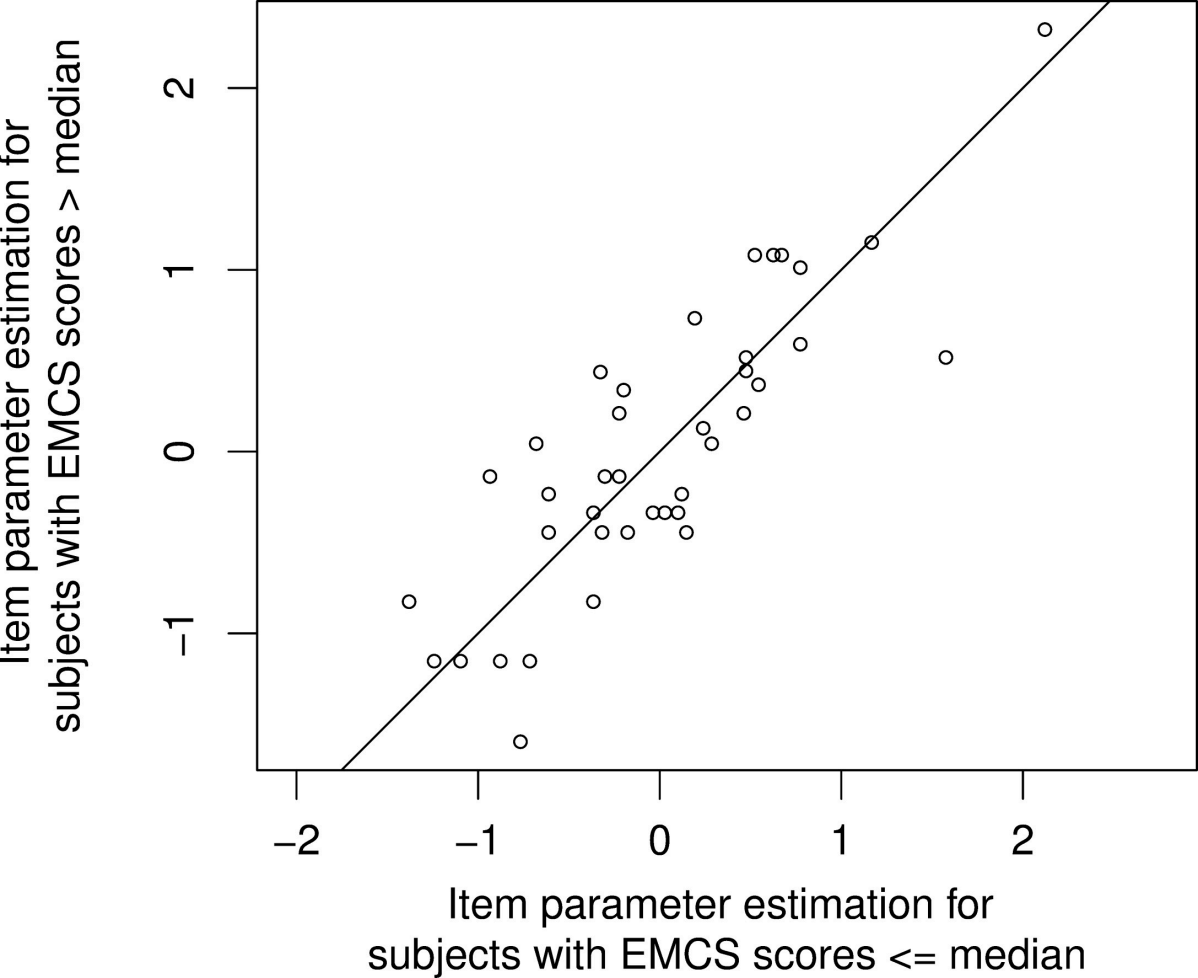
Graphical model check. Graphical model check plotting the item values estimated for participants with EMCS scores less or equal than the median against the item values estimated for participants with EMCS scores above the median. The results indicated a proper fit for all 40 items.

The Andersen test indicated a proper fit for the complete EMCS Scale (*LR*(39) = 45.49, *p* = 0.22), showing that there was one latent trait variable underlying our measures. Additionally, the graphical model test did not give hints for items, which fell out of line (see Fig 1). The item furthest away from the diagonal was item 24. However, this item still had an adequate mean-square outlier-sensitive fit of 0.82 [60], and it did also not show other striking item characteristics (see S2 Table for all item statistics and results of Rasch model analyses on single item basis).

There were neither indications against unidimensionality nor the valid fragmentation into the subsets A and B nor the further subdivision into socially close or socially distant protagonists (10 items each) in subsets A versus B (see Table 2). Moreover, the classical test theory fit index Cronbach’s alpha indicated a reasonable internal consistency for the total scale score as well as acceptable values for the subsets A and B. However, the additional split of set A and B into the 10-items parts depending on the social closeness of the protagonists showed rather low Cronbach’s alpha values, speaking against an uncoupled use of only these 10-items parts.

**Table 2:** Results of Martin-Loef LR-tests and classical test theory fit indices (Cronbach’s alpha) for the complete EMCS Scale, its two item sets A and B, and the additional split of the item sets A and B into each 10-items parts with socially close and socially distant protagonists.

	LR-value	df	*p*	Cronbach’s alpha
EMCS Scale (40 items)	166.69	398	1	0.84
Set A, set B (each 20 items)	141.40	399	1	0.60, 0.73
Set A socially close, set A socially distant, set B socially close, set B socially distant (each 10 items)	838.74	14597	1	0.37, 0.65, 0.58, 0.55

The descriptives of the inter-item correlations (*n* = 780, *M* = 0.11, *SD* = 0.15, *Min* = -0.34, *Max* = 0.75, skewness = 0.51, kurtosis = 0.99; for all details see S3 Table) and the item discriminations based on the complete EMCS Scale (*n* = 40, *M* = 0.23, *SD* = 0.08, *Min* = 0.09, *Max* = 0.43, skewness = 0.29, kurtosis = -0.61; for all details see S2 Table) indicated that the bivariate linearity of the items was not as strong as normally expected for dichotomous performance tests [61]. This might explain the somewhat lower values of Cronbach’s alpha, although the assumptions of the Rasch model were fulfilled.

### Effects of social closeness and gender on everyday moral decision-making

Concerning the social closeness of the protagonists, there were no significant differences in the percentage of altruistic decisions (Studies 1 and 3) and the ratings of altruistic responses (Study 2) between the items with socially close protagonists and the items with socially distant protagonists (see Table 3). However, there was a significant difference in the ratings of social closeness (Studies 1 and 2) in the expected direction with a very large effect size (*d* = 7.49), which additionally confirmed our *a priori* defined social closeness classifications.

**Table 3:** Means, standard deviations, and between-group comparisons regarding the percentage of altruistic decisions, altruistic and egoistic response ratings, social closeness ratings, and similarity to reality ratings separately for the 20 items with socially close and socially distant protagonists.

Variable			Socially close protagonists (20 items)		Socially distant protagonists (20 items)				
	*n*	Range	*M*	*SD*	*M*	*SD*	*t*	*p*	*d*
Percentage of altruistic decisions^1^	150	0–100	60.58	16.94	58.85	12.56	*t*(35.04) = 0.37	0.72	0.12
Altruistic response ratings^2^	50	1–7	5.70	0.30	5.83	0.46	*t*(32.93) = -1.11	0.27	0.36
Egoistic response ratings^2^	50	1–7	2.54	0.36	2.30	0.38	t(37.89) = 2.11	0.04	0.69
Social closeness^3^	100	1–7	5.85	0.77	1.77	0.19	*t*(21.39) = 23.10	<0.001	7.49
Similarity to reality^3^	100	1–7	4.39	0.46	4.94	0.51	*t*(37.63) = -3.57	0.001	1.16

^1^ Based on the available data from Studies 1 and 3

^2^ based on the available data from Study 2

^3^ based on the available data from Studies 1 and 2.

Additionally, the items with socially close protagonists differed from the items with socially distant protagonists regarding the ratings of egoistic responses (Study 2) and similarity to reality ratings (Studies 1 and 2; see Table 3). Egoistic responses of items with socially distant protagonists were rated as significantly more egoistic than egoistic responses of scenarios with socially close protagonists, indicating that fulfilling a personal desire towards strangers was perceived as slightly more egoistic than towards family and friends. Furthermore, the similarity to reality ratings were significantly higher for the items with socially distant protagonists, suggesting that our participants were more likely to encounter everyday moral conflict situations with strangers than with family or friends.

**Fig 2:**
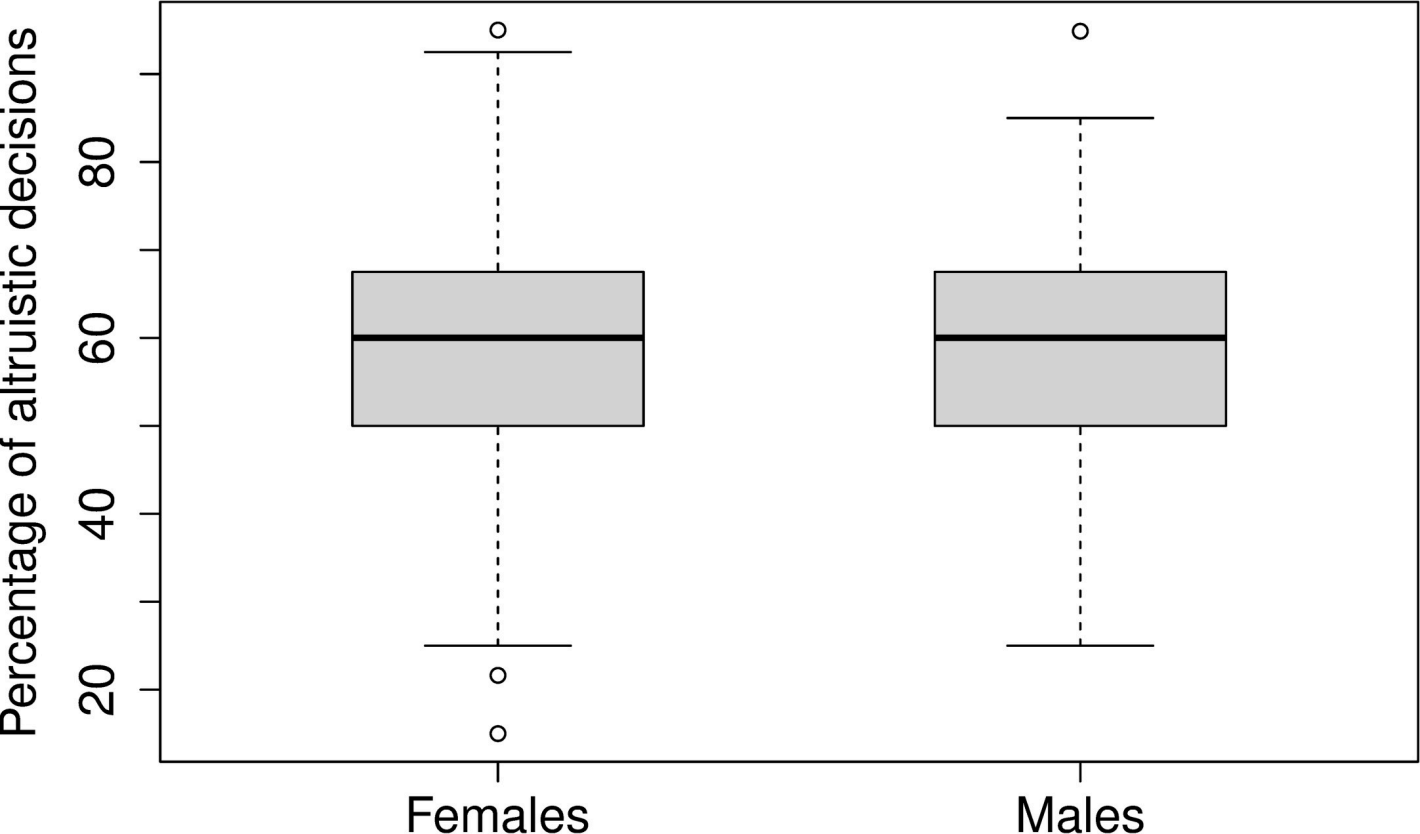
Boxplot for the percentage of altruistic decisions for females (*n* = 75) versus males (*n* = 75).

With regard to potential gender effects, there were no significant differences in the percentage of altruistic decisions between females (59.75% ± 15.53) and males (59.66% ± 13.68; *t*(145.67) = 0.04, *p* = 0.97, *d* = 0.01; see Fig 2), indicating that everyday moral decision-making did not differ depending on the gender of our participants.

### Supplementary analysis: Influence of the actual existence of socially close protagonists in the lives of participants

As a supplementary analysis, we finally investigated the impact of the actual existence of a respective socially close protagonist (i.e., partner, father, mother, brother, sister, uncle, aunt, grandfather, grandmother) in the lives of the participants on the percentage of altruistic decisions, social closeness ratings, and similarity to reality ratings. The performed analyses showed that there were no significant between-group differences in the percentage of altruistic decisions, social closeness ratings, or similarity to reality ratings (see Table 4), indicating that the actual existence of a respective socially close protagonist in the lives of participants had no impact on altruistic versus egoistic decision-making, social closeness ratings, or similarity to reality ratings in everyday moral conflict situations. It should be noticed that for the father and mother item, we were unable to perform between-group comparisons because all participants reported to have a father and mother who they knew or had known in the past. Moreover, for the uncle, aunt, grandfather, and grandmother item, we refrained from computing these analyses due to the partially very small and unequal sample sizes.

**Table 4:** Sample sizes and between-group comparisons (if applicable) regarding the impact of the actual existence of socially close protagonists in the lives of participants on the percentage of altruistic decisions, social closeness ratings, and similarity to reality ratings.

Socially close protagonist	Study	Actual existence in the lives of participants		Percentage of altruistic decisions^1^	Social closeness^2^	Similarity to reality^2^
		Yes	No			
Partner	1	25	25	*t*(47.98) = 1.71, *p* = 0.09, *d* = 0.48	*t*(86.92) = 0.23, *p* = 0.82, *d* = 0.05	*t*(97.76) = -0.14, *p* = 0.89, *d* = -0.03
	2	29	21			
Father	1	50	0	-	-	-
	2	50	0			
Mother	1	50	0	-	-	-
	2	50	0			
Brother	1	31	19	*t*(35.52) = 0.81, *p* = 0.43, *d* = 0.26	*t*(57.17) = 1.05, *p* = 0.30, *d* = 0.24	*t*(86.36) = -0.85, *p* = 0.40, *d* = -0.17
	2	30	20			
Sister	1	31	19	*t*(40.00) = -0.82, *p* = 0.42, *d* = -0.22	*t*(97.16) = -0.43, *p* = 0.67, *d* = -0.09	*t*(90.47) = 0.75, *p* = 0.46, *d* = 0.15
	2	27	23			
Uncle	1	48	2	-	-	-
	2	43	7			
Aunt	1	48	2	-	-	-
	2	46	4			
Grandfather	1	48	2	-	-	-
	2	46	4			
Grandmother	1	50	0	-	-	-
	2	49	1			

^1^ Based on the available data from Study 1

^2^ based on the available data from Studies 1 and 2.

## Discussion

Despite the growing field of moral psychology [62], there is still a paucity of validated everyday moral decision-making paradigms for use in laboratory settings. Therefore, we conducted three independent studies to develop and validate a new measure to assess decision-making in everyday moral conflict situations. To sum up, our new EMCS Scale, which can be applied both as paper-pencil questionnaire and as computer task, comprises 40 everyday moral conflict scenarios with high similarity to everyday life situations. Concerning the content of our scenarios, we got inspired by the MFT [19–21] as well as the empirical results of a large ecological momentary assessment study [18]. Instead of varying the emotionality of the stories as done in previous research (e.g., high vs. low emotionality; [22, 23]), we varied the social closeness of the protagonists (socially close vs. socially distant) in order to investigate the impact of the relatedness of the participants to the story characters as a further potential modulating factor of everyday moral decision-making [2]. Additionally, we counterbalanced our new measure in terms of the gender of the protagonists and equally recruited males and females for participation in our surveys to explore potential gender differences. For the first time in everyday moral dilemma research, we additionally developed and validated two parallelized item sets for future use in within-subjects design studies. This offers the opportunity to control for potential effects of interindividual differences in trait variables (e.g., personality; see [26]).

During the process of scale development, we selected 40 out of 60 items with preferable mean rates of altruistic decisions in order to avoid ceiling or floor effects. This is in contrast to earlier studies (e.g., [22]), where especially high-emotional dilemmas came along with a very high percentage of altruistic decisions (up to 96%). Moreover, we only selected items with a clear representation of altruistic and egoistic response classes, unambiguous social closeness classifications, and high similarity to reality ratings. Regarding our final 40-items set, all items provided satisfactory psychometric parameters (percentage of altruistic decisions between 17% and 83%, altruistic response ratings above and egoistic response ratings below the mean scale value of 4, social closeness ratings for items with socially close protagonists above and for items with socially distant protagonists below the mean scale value of 4, and relatively high similarity to reality ratings; see Tables 3 and S1). Additionally, using Wilks L_mvc_ tests [54], we successfully developed two parallelized 20-items sets A and B.

With regard to the test and measurement properties, our results showed that both the EMCS Scale and its two subsets A and B fitted the Rasch model, which implied that there was one underlying latent trait variable. Furthermore, the classical test theory fit index Cronbach’s alpha indicated reasonable internal consistencies for the total EMCS Scale as well as the two item sets A and B (0.60 ≤ *α* ≥ 0.84). Even the fragmentation into four parts still resulted in a sufficient estimation by the Rasch model, although Cronbach’s alpha results spoke against an uncoupled use of only these 10-items parts. Thus, both the complete EMCS Scale and its two parallelized subsets A and B can be utilized as valid measures for decision-making in everyday moral conflict situations.

In our data, we did not observe that the percentage of altruistic decisions differed depending on the social closeness of the protagonists. This is in contrast to the results of Zhan and colleagues [31], the only study so far that investigated the impact of social closeness on everyday moral decision-making. Contrary to Zhan et al. [31], we only observed a slightly, but not significantly lower percentage of altruistic decisions for the items with socially distant protagonists than for the items with socially close protagonists, and this difference only reached a very small effect size (*d* = 0.12). Additionally, Rasch model analyses indicated that there was one underlying latent trait variable, which further speaks against a significant impact of social closeness on everyday moral decision-making in our surveys. This divergent finding could possibly be explained by methodological differences. Since Zhan et al. [31] did not provide concrete examples of their stimulus material in their manuscript, it remains unclear whether their vignettes represented everyday moral conflict situations. Furthermore, our data also appear to be in contrast to several abstract moral decision-making studies, which showed that social closeness is an important experimental design parameter in moral dilemma research ([5, 28–30]; see also [2]). One potential explanation could be that abstract moral dilemmas describe dead-or-alive situations, whereas the consequences of the response alternatives in our everyday moral dilemmas are less grave. Therefore, one might be more willing to accept the costs of an egoistic response option not only for socially distant others, but also to some degree for socially close persons.

Somewhat unexpectedly, we found that the egoistic responses of items with socially distant protagonists were rated as significantly more egoistic than the egoistic responses of items with socially close protagonists. We also observed this group difference for the altruistic response ratings, at least on a descriptive level and with a medium effect size (*d* = 0.36; i.e., altruistic responses of items with socially distant protagonists were rated as slightly, but not significantly more altruistic than altruistic responses of items with socially close protagonists). These interesting results could be potentially traced back to the fact that the similarity to reality ratings were also significantly higher for the scenarios with socially distant protagonists, indicating that our participants are more likely to encounter everyday moral conflict situations with strangers than with family or friends. This is in line with the results of a recent ecological momentary assessment study about morality in everyday life, where the most frequent type of victim categories were “no concrete person/other entity” or “stranger” [17]. Taken together, these findings combined with our data raise the hypothesis that participants might be better able to evaluate more frequently experienced everyday moral conflict situations, namely conflicts with socially distant than with socially close others, and consequently are more confident to choose the more extreme response categories.

As a supplementary analysis, we further examined if the responses to the items with socially close protagonists differed depending on the actual existence of the respective target persons in the real lives of the participants. The rationale behind this investigation was to rule out the possibility that the actual existence, respectively non-existence, of the socially close protagonists in the lives of participants might be a potential confounding factor that influences decision-making in everyday moral conflict situations. This appeared not to be the case, since we did not observe any between-group differences in the percentage of altruistic decisions, social closeness ratings, and similarity to reality ratings (as far as the analyses were feasible; for details see the results section). Thus, our results provide evidence that the EMCS Scale can be broadly applied independently of the actual existence of the respective persons in participants’ lives.

Interestingly, we also did not observe gender differences in our surveys, which, on the one hand, is not uncommon for hypothetical moral dilemmas [43], but, on the other hand, is in sharp contrast to early moral reasoning research [42] and current abstract moral decision-making studies [7, 38–41]. This inconsistent result could potentially be explained by the fact that our new EMCS Scale measures altruistic and egoistic response tendencies, which are behavioral measures that rather reflect outcomes of morality, but not moral attitudes itself [43]. Altogether, in combination with the social closeness results, our data therefore point to the idea that everyday moral decision-making with altruistic versus egoistic response options seems to be quite a different construct than abstract moral decision-making with utilitarian versus deontological response alternatives.

One obvious limitation of our new measurement tool is that the EMCS Scale, which was developed for use in laboratory settings, cannot reach external and ecological validity as high as ecological momentary assessment (e.g., [17, 18]). However, the similarity to reality ratings achieved in our surveys showed that all selected stories may at least potentially occur in everyday life, thus indicating relatively high ecological validity of our new scale. Moreover, our participants chose the altruistic response alternatives in almost 60% of all cases, which is in accordance with the fact that a remarkable facet of human behavior is that people often decide to help others even when it comes at personal cost, and when there is no expectation of receiving any material returns [63]. It should also be considered that research regarding morality in everyday life is still in its infancies in many application fields (e.g., regarding the impact of stress and stress hormones on everyday moral decision-making; see [24]). Hence, in a first step, controlled laboratory studies with measures like the EMCS Scale are important to achieve higher internal validity.

Furthermore, we did not explicitly control for socially desirable responding in our surveys. Nevertheless, we tried to keep the potential impact of social desirability as low as possible by ensuring strict anonymity to all our participants. Moreover, we excluded all items with extremely high percentages of altruistic decisions. For the remaining items, we observed statistical variance both within participants and across items, which probably speaks against highly socially desirable responding.

Additionally, it has to be acknowledged that we did not achieve an equal distribution of the four moral dimensions (namely care/harm, fairness/unfairness, loyalty/disloyalty, and honesty/dishonesty) as introduced by MFT. However, to date, it is still a question of debate whether morality is a unified construct or can be deconstructed into several factors [62, 64]. Our data seem to provide support for the assumption of unidimensionality (one latent trait variable). For future studies, a much larger data base would be necessary to perform a further in-depth analysis of the potentially underlying factoral structure. In this regard, we would expect a bifactor structure (i.e., loadings on both a global and several specific factors), since in the EMCS Scale, a clear distinction of different moral dimensions (as for example realized by [65]) had to stand back from the aim of high criterion validity for the everyday life situations. Indeed, explorative factor analysis of the present data suggested at least six factors, thus underscoring the idea that there might be a somewhat more complicated underlying measurement structure. But, as Reise et al. [66] summarized, there would be a need of much bigger sample sizes to perform such analyses. Moreover, for future research, it would be interesting to examine gender differences in the response behavior to the EMCS Scale in more detail (e.g., regarding the impact of the gender of the participants depending on the gender of the protagonists; see [67] or [68] for examples in other research areas). Additionally, it would be conceivable that social closeness as an important experimental design parameter in everyday moral decision-making may only emerge in combination with certain experimental manipulations, like the induction of acute psychosocial stress (e.g., [33]; for a potential theoretical explanation see [69]). Therefore, we highly encourage researchers to use the EMCS Scale to explore the impact of social closeness on everyday moral decision-making in combination with different experimental paradigms and tasks. More generally, future studies should also try to develop measures for the investigation of other types of moral conflicts in everyday life situations (e.g., conflicts between different duties or between sets of apparently incommensurable values; see [2]), since the EMCS Scale covers only one particular sort of moral conflicts, namely accepted moral values versus self-interests.

In conclusion, our new EMCS Scale appears to be a promising new measurement tool for the investigation of the manifold determinants of decision-making in everyday moral conflict situations. In particular, we developed and validated a measure that is composed of two parallelized item sets A and B. Therefore, it can be utilized both in between- and within-subjects design studies. In between-subjects design studies, the EMCS Scale offers the opportunity to realize more than one point of measurement (e.g., at different time points before and after an experimental/control manipulation). In within-subjects design studies, the parallelized item sets provide the advantage of controlling for interindividual variability in trait variables affecting everyday moral decision-making. Altogether, the EMCS Scale may prove useful in many areas of application, as for instance in individual differences research, social psychology, psychobiological stress research, clinical psychology, or occupational and organizational psychology.

## Supporting information

S1 Table The EMCS scale. S1 Table shows the finally selected 40 items (everyday moral conflict situations) of the EMCS Scale with corresponding response alternatives (altruistic vs. egoistic), mean item difficulties (Studies 1 and 3), altruistic and egoistic response ratings (Study 2), social closeness and similarity to reality ratings (Studies 1 and 2), and respective assignment to the two parallelized item sets A and B.
(DOCX)

S2 Table Item statistics and results of Rasch model analyses. S2 Table shows the item statistics and results of Rasch model analyses on single item basis for the final 40 items of the EMCS Scale.
(DOCX)

S3 Table Inter-item correlations. S3 Table shows the inter-item correlations (tetrachoric correlations) of the final 40 items of the EMCS Scale.
(DOCX)

S1 File SPSS data of Studies 1 and 2. Based on this data set (*n* = 100), we selected 40 out of the initially 60 items with a preferable mean rate of altruistic decisions, a clear representation of altruistic and egoistic response classes, unambiguousness of social closeness classifications, and high similarity to reality ratings.
(SAV)

S2 File SPSS data of Studies 1 and 3. Based on this data set (*n* = 150), we developed the two parallelized item sets for future use in within-subjects design studies and assessed the test and measurement properties of the EMCS Scale.
(SAV)
