# Supplementary material for: Decision-making in everyday moral conflict situations: Development and validation of a new measure
Source: PLoS One. 2019 Apr 1;14(4):e0214747. doi: 10.1371/journal.pone.0214747 (PMC6443167; doi:10.1371/journal.pone.0214747)
Supplement: S1 Table — shows the finally selected 40 items (everyday moral conflict situations) of the EMCS Scale with corresponding response alternatives (altruistic vs. egoistic), mean item difficulties (Studies 1 and 3), altruistic and egoistic response ratings (Study 2), social closeness and similarity to reality ratings (Studies 1 and 2), and respective assignment to the two parallelized item sets A and B. (DOCX) [file pone.0214747.s001.docx]

**S1 Table. The EMCS Scale.**

Overview of the finally selected 40 items (everyday moral conflict situations) of the EMCS Scale with corresponding response alternatives (altruistic vs. egoistic), mean item difficulties (Studies 1 and 3), altruistic and egoistic response ratings (Study 2), social closeness and similarity to reality ratings (Studies 1 and 2), and respective assignment to the two parallelized item sets A and B. Items 1-20 are scenarios with socially close protagonists, items 21-40 are scenarios with socially distant protagonists.

| **Everyday moral conflict situation** | **Response alternatives (altruistic vs. egoistic)** | **Item difficulty  (0-1)^1^** | **Altruistic response rating (1-7)^2^** | **Egoistic response rating (1-7)^2^** | **Social closeness (1-7)^3^** | **Similarity  to reality (1-7)^3^** | **Set (A/B)** | | |
| --- | --- | --- | --- | --- | --- | --- | --- | --- | --- |
| ***Items with socially close protagonists*** | | | | | | | |  |  |
| 1. I buy concert tickets on the internet for my brother and myself. I can get two seats side by side. However, the tickets fall into two different price categories, so that one ticket is 10 € more expensive than the other. What do I do? | I take the more expensive ticket.  I take the cheaper ticket. | 0.61 (± 0.49) | 5.72 (± 1.02) | 2.56 (± 1.08) | 6.73 (± 0.62) | 4.03 (± 1.43) | B | | |
| 1. I am just about to leave for work as a neighbor and friend rings my bell. She asks me if I could drive her to the doctor because she is not feeling well. I am already late and have a meeting with my boss today. What do I do? | I drive her to the doctor.  I go to work. | 0.50 (± 0.50) | 6.12 (± 0.97) | 2.60 (± 0.91) | 4.75 (± 0.78) | 4.10 (± 1.24) | A | | |
| 1. Today, it is games night and I play poker for money with friends. As one of my opponents shortly gets distracted, I catch a glance of his/her cards. The other players do not notice anything. What do I do? | I let the cards be reshuffled.  I keep playing. | 0.17 (± 0.37) | 5.80 (± 0.91) | 2.84 (± 1.07) | 5.61 (± 0.84) | 4.77 (± 1.57) | B | | |
| 1. I have been single for quite a long time. Tonight, I finally have a promising date again. As I am about to leave, a good friend of mine who is not feeling well gives me a call and wants to meet up with me now. What do I do? | I meet up with my friend.  I go on the date. | 0.66 (± 0.48) | 5.40 (± 1.08) | 2.48 (± 0.82) | 6.04 (± 0.94) | 4.88 (± 1.54) | B | | |
| 1. A friend of mine definitely wants to buy an old computer game from me that he loves playing. I promise to sell it to him for 20 € tomorrow. Later on, as I check on the internet, I see that I could sell the game immediately for 60 € there. What do I do? | I keep my promise.  I sell the game for more money. | 0.79 (± 0.41) | 5.64 (± 1.00) | 2.40 (± 0.82) | 5.63 (± 0.75) | 4.14 (± 1.54) | A | | |
| 1. I am talking to my aunt at a family celebration. All of a sudden, it comes to my mind that she lent me a CD last Christmas. She does not seem to remember anymore and I like the CD very much. What do I do? | I return the CD.  I keep the CD. | 0.74 (± 0.44) | 5.44 (± 1.00) | 2.12 (± 1.01) | 5.70 (± 0.96) | 4.78 (± 1.47) | B | | |
| 1. I have promised my sister to take care of her children tonight. Now, I realize that I am also invited to a farewell party today that is very important for me. I could think up an excuse to go to the party. What do I do? | I keep my promise.  I think up an excuse. | 0.70 (± 0.46) | 5.28 (± 0.98) | 2.16 (± 1.03) | 6.71 (± 0.57) | 4.80 (± 1.52) | A | | |
| 1. A friend of mine has inherited a coin collection that interests me very much. He offers to sell the coins to me for a low price. The coins are already collector’s items and would therefore actually be worth considerably more money. What do I do? | I inform the friend about the value.  I buy the coins for the low price. | 0.62 (± 0.49) | 5.76 (± 0.72) | 3.28 (± 1.14) | 5.65 (± 0.86) | 3.67 (± 1.51) | A | | |
| 1. A friend of mine and I are big fans of a band. This band is going to give a concert in our hometown, and we both want to go. At the ticket agency, I am only able to get one ticket. What do I do? | I give the ticket to my friend.  I go to the concert myself. | 0.62 (± 0.49) | 6.32 (± 0.95) | 2.64 (± 1.08) | 5.82 (± 0.74) | 3.68 (± 1.49) | A | | |
| 1. While locking up my bike, it falls against a car. In the darkness, I do not detect any scratches on the car. The next day, I hear my well-known neighbor complaining about a fresh scratch on his new car. What do I do? | I inform the neighbor.  I keep quiet about the incident. | 0.56 (± 0.50) | 5.68 (± 0.99) | 2.08 (± 0.76) | 4.27 (± 1.18) | 4.48 (± 1.31) | A | | |
| 1. It is the soccer world cup and the final match is on TV. I am a big soccer fan and very excited about the game. All of a sudden, a friend of mine who is not feeling well gives me a call and wants to meet up with me right now. What do I do? | I meet up with my friend.  I watch the soccer game. | 0.81 (± 0.39) | 5.60 (± 0.71) | 2.44 (± 1.61) | 5.76 (± 1.00) | - 1. ± 1.80) | A | | |
| 1. I have promised my partner to go to the company party with him/her. He/she has already signed both of us up. Now I realize that I would urgently need the time to prepare for an important exam. What do I do? | I keep my promise.  I prepare for the exam. | 0.51 (± 0.50) | 5.68 (± 0.95) | 3.12 (± 1.05) | 6.65 (± 0.82) | 4.89 (± 1.53) | A | | |
| 1. My mother gives me 20 € to buy pet food. At the pet store, I see that the pet food is on special offer and only costs 10 €. Since I have many expenses this month, I could use the remaining 10 € very well for myself. What do I do? | I return the money to my mother.  I keep the money. | 0.77 (± 0.42) | 5.48 (± 1.01) | 2.36 (± 1.08) | 6.75 (± 0.77) | 5.11 (± 1.45) | B | | |
| 1. A good band is going to give a concert in town. At the ticket agency, I get the very last ticket. When a classmate and friend standing behind me in line realizes this, he is appalled and bursts into tears. What do I do? | I give the ticket to him.  I keep the ticket for myself. | 0.61 (± 0.49) | 5.96 (± 1.06) | 2.88 (± 1.27) | 4.85 (± 0.97) | 3.57 (± 1.55) | B | | |
| 1. I have promised my grandfather to help him complete urgent forms this evening. All of a sudden, I get a phone call from the newspaper. I have won tickets for my favorite band’s sold out concert tonight. What do I do? | I help my grandfather.  I go to the concert. | 0.45 (± 0.50) | 6.12 (± 0.73) | 2.72 (± 1.06) | 6.72 (± 0.81) | 3.80 (± 1.58) | B | | |
| 1. I am at the airport, ready to leave on a long-planned holiday. While I am standing at the check-in counter, my mother gives me a call. She tells me that my father had a little accident and was admitted to the hospital. What do I do? | I cancel the holiday.  I take the flight anyway. | 0.41 (± 0.49) | 5.88 (± 1.05) | 2.24 (± 1.17) | 6.72 (± 0.89) | 4.47 (± 1.45) | A | | |
| 1. I have promised a neighbor and friend to receive an urgent parcel for her today. Now, it is 5 pm and the parcel service has not been here yet. My gym course will start soon and I would really like to participate. What do I do? | I wait for the parcel service.  I go to the gym course. | 0.43 (± 0.50) | 6.00 (± 0.91) | 3.16 (± 0.99) | 4.74 (± 0.94) | 4.86 (± 1.38) | B | | |
| 1. I have promised to go to a friend’s birthday party. Now, I realize that it is the same day as my favorite band’s concert, which I do not want to miss. I could think up an excuse to go to the concert. What do I do? | I go to the birthday party.  I think up an excuse. | 0.83 (± 0.38) | 5.24 (± 0.97) | 2.20 (± 0.87) | 5.56 (± 1.00) | - 1. ± 1.34) | B | | |
| 1. I want to sell my old laptop. As my uncle hears about it, he offers to pay 200 € and I agree. Soon after, I see on an internet portal that I could sell the laptop immediately for 300 € there. What do I do? | I keep my promise.  I sell the laptop for more money. | 0.83 (± 0.38) | 5.32 (± 1.07) | 2.28 (± 0.68) | 5.75 (± 0.83) | - 1. ± 1.37) | B | | |
| 1. I have promised my grandmother to take her to the doctor this afternoon. One hour before the appointment, my boss gives me a call and summons me on short notice for an important meeting. This meeting is supposed to be about my promotion. What do I do? | I take my grandmother to the doctor.  I go to the meeting with my boss. | 0.52 (± 0.50) | 5.48 (± 1.09) | 2.32 (± 0.69) | 6.48 (± 0.82) | 4.32 (± 1.38) | A | | |
| ***Items with socially distant protagonists*** | | | | | | | | |  |
| 1. While pulling out of a supermarket parking space, I accidentally bump the car parking next to mine. At first glance, I cannot detect any damages on my car. It is already dark and nobody else has seen anything. What do I do? | I leave a message for the owner of the car.  I drive away quickly. | 0.79 (± 0.41) | 5.24 (± 1.36) | 1.56 (± 0.77) | 1.42 (± 0.93) | 5.73 (± 1.16) | B | | |
| 1. After some hours in a café, I request to pay. I have already calculated the total amount to be paid in my head. When the waitress tells me the amount to be paid, I realize that she has miscalculated by 3 € in my favor. What do I do? | I return the money.  I keep the money | 0.61 (± 0.49) | 6.25 (± 0.99) | 1.84 (± 0.69) | 2.23 (± 1.05) | 5.55 (± 1.20) | B | | |
| 1. I want to go home on a train that runs only once every hour. The train is about to leave and I am just boarding as a man with crutches near me falls on the platform. If I help him up, I will miss the train. What do I do? | I help the man.  I take the train. | 0.71 (± 0.46) | 6.32 (± 0.85) | 1.84 (± 0.90) | 1.80 (± 1.03) | 4.76 (± 1.30) | B | | |
| 1. After a visit to a cocktail bar during my holiday, I request to pay. A waiter brings me the bill. While looking over it, I notice that he forgot to bill my last cocktail. What do I do? | I point out the mistake.  I pay without the last cocktail. | 0.37 (± 0.48) | 5.80 (± 0.76) | 2.52 (± 0.87) | 1.92 (± 0.88) | 5.37 (± 1.03) | A | | |
| 1. I advertise my old computer for sale on the internet. A potential buyer and I agree on a price of 320 €. However, the buyer accidentally transfers 40 € too much to my bank account. What do I do? | I transfer the money back.  I keep the money. | 0.68 (± 0.47) | 5.92 (± 0.76) | 2.08 (± 0.86) | 1.81 (± 1.03) | 4.38 (± 1.38) | B | | |
| 1. I want to sell my old car. I know that the car’s radiator actually needs to be exchanged urgently. A man who does not notice the problem with the radiator offers to pay a good price in cash right away. What do I do? | I mention the defect.  I keep quiet about the defect. | 0.72 (± 0.45) | 5.88 (± 0.83) | 1.68 (± 0.69) | 1.87 (± 0.92) | 4.69 (± 1.41) | A | | |
| 1. I am about to get into my car at a supermarket parking lot. Next to me, a woman’s full bag of groceries bursts and all her purchases fall to the ground. If I help the woman, I will be too late for an important appointment. What do I do? | I help the woman.  I get into my car. | 0.68 (± 0.47) | 6.32 (± 0.69) | 2.08 (± 0.86) | 1.83 (± 0.95) | 5.09 (± 1.28) | A | | |
| 1. I have placed an advertisement to sell a wardrobe. A woman who wants to pick up the wardrobe next week offers to pay 140 € and I agree. Soon after, someone else calls who offers to pay 200 €. What do I do? | I keep my promise.  I sell the wardrobe for more money. | 0.49 (± 0.50) | 5.64 (± 0.81) | 2.80 (± 1.08) | 1.75 (± 0.99) | 4.86 (± 1.36) | B | | |
| 1. I want to get on a bus that is about to leave and that only runs once every 30 minutes. On the bus platform, there is an old woman with a bag of groceries. As I am getting on the bus, the woman’s bag falls to the ground. What do I do? | I help the woman.  I take the bus. | 0.60 (± 0.49) | 6.40 (± 0.65) | 2.44 (± 0.82) | 1.81 (± 0.99) | 5.00 (± 1.35) | B | | |
| 1. I am at the checkout of a supermarket and I want to pay for my groceries, which cost 8 €. I give a 10 € bill to the cashier. She accidentally gives me back 4 € instead of 2 €. What do I do? | I return the money.  I keep the money. | 0.62 (± 0.49) | 5.80 (± 0.91) | 2.52 (± 1.23) | 1.91 (± 1.07) | 5.67 (± 1.13) | A | | |
| 1. I am running to catch a bus that is about to leave and that only runs once every hour. In front of me, several items drop out of the purse of a woman with two small children. Except for me, there is no one else around to help the woman. What do I do? | I help the woman.  I run to the bus. | 0.61 (± 0.49) | 5.96 (± 0.79) | 2.16 (± 1.03) | 1.80 (± 1.03) | 4.97 (± 1.34) | A | | |
| 1. After a visit to the shopping center, I am on my way back to my car. By chance, I find a 20 € bill on the ground. As I look around, I notice a homeless man going through the dustbins of the shopping center. What do I do? | I give the money to the homeless man.  I keep the money. | 0.41 (± 0.49) | 6.52 (± 0.87) | 2.72 (± 1.17) | 1.47 (± 0.88) | 3.83 (± 1.54) | B | | |
| 1. I got the confirmation for a terrific apartment. However, the landlord does not permit pets, and I own a hamster. The landlord lives 100 km away from the apartment and would probably never find out about the pet. What do I do? | I do not take the apartment.  I keep quiet about the pet. | 0.42 (± 0.50) | 4.88 (± 1.20) | 2.84 (± 0.99) | 1.96 (± 1.02) | 4.76 (± 1.39) | B | | |
| 1. I have ordered a pizza from a delivery service on the internet. After delivery, I pay with a 50 € bill. As the pizza delivery boy gives me the change, I realize that he has given me back 5 € too much. What do I do? | I return the money.  I keep the money. | 0.65 (± 0.48) | 5.36 (± 0.91) | 2.24 (± 0.93) | 1.83 (± 1.06) | 5.26 (± 1.13) | B | | |
| 1. I want to sell a painting at a flea market. A woman offers to pay 100 € and I agree. While the woman is on her way to a bank to withdraw money, someone else offers to pay 150 € for the painting. What do I do? | I keep my promise.  I sell the painting for the higher price. | 0.71 (± 0.46) | 5.60 (± 1.00) | 2.52 (± 1.09) | 1.76 (± 0.93) | 4.33 (± 1.39) | A | | |
| 1. On the street, I see an old woman stumble and her purchases roll on the ground. Next to me, I see my bus that is about to leave and that runs only once every two hours. Besides the woman, I am the only person around. What do I do? | I help the woman.  I take the bus. | 0.56 (± 0.50) | 6.00 (± 0.96) | 2.56 (± 1.12) | 1.75 (± 1.05) | 4.29 (± 1.42) | B | | |
| 1. I find a wallet on the street one evening with 50 € in it but without any personal documents. There is no possibility for me to find out the owner. However, I could turn in the wallet at the city’s lost and found office. What do I do? | I turn in the wallet.  I keep the wallet. | 0.66 (± 0.48) | 6.12 (± 0.88) | 2.04 (± 0.84) | 1.47 (± 1.28) | 4.80 (± 1.54) | A | | |
| 1. I am driving by car to an important business meeting and I am running a bit late today. Right in front of me, a slight rear-end collision happens. If I stop my car, I will probably be too late for my meeting. What do I do? | I stop my car.  I keep driving. | 0.50 (± 0.50) | 5.48 (± 1.05) | 2.52 (± 1.01) | 1.48 (± 0.86) | 4.94 (± 1.44) | A | | |
| 1. I definitely want to catch the bus in order to be home in time for an important appointment. Shortly before the bus leaves, the pedestrian light turns red. A little boy is standing on the other side of the intersection. What do I do? | I wait.  I cross the street on red light. | 0.35 (± 0.48) | 5.04 (± 1.02) | 2.72 (± 1.10) | 1.78 (± 1.17) | 5.74 (± 1.11) | A | | |
| 1. I want to go home by train. As I am getting on the train, I see a man with crutches unsuccessfully trying to carry his suitcase upstairs to the platform. If I help the man, I will miss the train. What do I do? | I help the man.  I get on the train. | 0.63 (± 0.48) | 6.12 (± 0.93) | 2.28 (± 0.98) | 1.77 (± 1.11) | 4.83 (± 1.45) | A | | |

^1^ Based on the available data from 150 participants (Studies 1 and 3); ^2^ based on the available data from 50 participants (Study 2); ^3^ based on the available data from 100 participants (Studies 1 and 2); the 40 items were presented in a fixed random order in all three studies; the order of the two corresponding response alternatives was counterbalanced in our surveys.
