# Supplementary material for: Decision-making in everyday moral conflict situations: Development and validation of a new measure
Source: PLoS One. 2019 Apr 1;14(4):e0214747. doi: 10.1371/journal.pone.0214747 (PMC6443167; doi:10.1371/journal.pone.0214747)
Supplement: S2 Table — shows the item statistics and results of Rasch model analyses on single item basis for the final 40 items of the EMCS Scale. (DOCX) [file pone.0214747.s002.docx]

**S2 Table. Item statistics and results of Rasch model analyses.**

Item statistics and results of Rasch model analyses on single item basis for the final 40 items of the EMCS Scale. Items 1-20 are scenarios with socially close protagonists, items 21-40 are scenarios with socially distant protagonists.

|  | **Item statistics** | | | | | | | | |  | | | **Rasch model analyses** | | | | | | | | | | | | |  |
| --- | --- | --- | --- | --- | --- | --- | --- | --- | --- | --- | --- | --- | --- | --- | --- | --- | --- | --- | --- | --- | --- | --- | --- | --- | --- | --- |
|  | | Item difficulty | | Sample *SD* | | Discrimination: Item with total score | | Discrimination: Item with total score without CIS | | |  | | | Beta | | *SE* | | Outfit *MSQ* | | Infit *MSQ* | | Outfit *t* | | Infit *t* | |  |
| Item 1 | | | 0.58 | | 0.50 | | 0.28 | | 0.20 | | |  | | | -0.09 | | 0.17 | | 1.03 | | 1.03 | | 0.44 | | 0.60 | |
| Item 2 | | | 0.49 | | 0.50 | | 0.31 | | 0.23 | | |  | | | -0.47 | | 0.17 | | 1.03 | | 1.01 | | 0.37 | | 0.31 | |
| Item 3 | | | 0.17 | | 0.37 | | 0.34 | | 0.28 | | |  | | | -2.20 | | 0.22 | | 0.90 | | 0.89 | | -0.47 | | -0.75 | |
| Item 4 | | | 0.65 | | 0.48 | | 0.27 | | 0.20 | | |  | | | 0.24 | | 0.18 | | 1.02 | | 1.02 | | 0.19 | | 0.33 | |
| Item 5 | | | 0.79 | | 0.41 | | 0.27 | | 0.20 | | |  | | | 0.96 | | 0.20 | | 0.96 | | 1.00 | | -0.16 | | 0.00 | |
| Item 6 | | | 0.74 | | 0.44 | | 0.26 | | 0.19 | | |  | | | 0.69 | | 0.19 | | 1.06 | | 1.00 | | 0.48 | | 0.00 | |
| Item 7 | | | 0.69 | | 0.46 | | 0.26 | | 0.18 | | |  | | | 0.44 | | 0.18 | | 1.03 | | 1.02 | | 0.29 | | 0.28 | |
| Item 8 | | | 0.62 | | 0.49 | | 0.28 | | 0.20 | | |  | | | 0.09 | | 0.17 | | 1.01 | | 1.03 | | 0.08 | | 0.48 | |
| Item 9 | | | 0.59 | | 0.49 | | 0.17 | | 0.09 | | |  | | | -0.03 | | 0.17 | | 1.14 | | 1.10 | | 1.58 | | 1.85 | |
| Item 10 | | | 0.56 | | 0.50 | | 0.36 | | 0.28 | | |  | | | -0.18 | | 0.17 | | 0.94 | | 0.97 | | -0.82 | | -0.59 | |
| Item 11 | | | 0.80 | | 0.40 | | 0.27 | | 0.20 | | |  | | | 1.05 | | 0.21 | | 0.99 | | 0.98 | | -0.02 | | -0.15 | |
| Item 12 | | | 0.51 | | 0.50 | | 0.25 | | 0.16 | | |  | | | -0.39 | | 0.17 | | 1.05 | | 1.06 | | 0.70 | | 1.28 | |
| Item 13 | | | 0.77 | | 0.42 | | 0.24 | | 0.17 | | |  | | | 0.84 | | 0.20 | | 0.95 | | 1.04 | | -0.25 | | 0.39 | |
| Item 14 | | | 0.61 | | 0.49 | | 0.22 | | 0.14 | | |  | | | 0.06 | | 0.17 | | 1.11 | | 1.06 | | 1.19 | | 1.10 | |
| Item 15 | | | 0.45 | | 0.50 | | 0.43 | | 0.35 | | |  | | | -0.68 | | 0.17 | | 0.89 | | 0.92 | | -1.53 | | -1.52 | |
| Item 16 | | | 0.41 | | 0.49 | | 0.26 | | 0.17 | | |  | | | -0.86 | | 0.17 | | 1.05 | | 1.07 | | 0.69 | | 1.11 | |
| Item 17 | | | 0.43 | | 0.50 | | 0.25 | | 0.16 | | |  | | | -0.74 | | 0.17 | | 1.14 | | 1.05 | | 1.77 | | 0.95 | |
| Item 18 | | | 0.83 | | 0.38 | | 0.20 | | 0.14 | | |  | | | 1.23 | | 0.22 | | 0.98 | | 1.02 | | -0.03 | | 0.22 | |
| Item 19 | | | 0.83 | | 0.38 | | 0.28 | | 0.22 | | |  | | | 1.23 | | 0.22 | | 0.92 | | 0.95 | | -0.36 | | -0.32 | |
| Item 20 | | | 0.51 | | 0.50 | | 0.35 | | 0.27 | | |  | | | -0.39 | | 0.17 | | 1.01 | | 0.97 | | 0.11 | | -0.54 | |
| Item 21 | | | 0.78 | | 0.42 | | 0.31 | | 0.25 | | |  | | | 0.92 | | 0.20 | | 0.95 | | 0.96 | | -0.24 | | -0.32 | |
| Item 22 | | | 0.61 | | 0.49 | | 0.40 | | 0.33 | | |  | | | 0.06 | | 0.17 | | 0.88 | | 0.94 | | -1.33 | | -1.11 | |
| Item 23 | | | 0.71 | | 0.46 | | 0.39 | | 0.32 | | |  | | | 0.51 | | 0.18 | | 0.86 | | 0.94 | | -1.17 | | -0.79 | |
| Item 24 | | | 0.37 | | 0.48 | | 0.50 | | 0.43 | | |  | | | -1.04 | | 0.18 | | 0.82 | | 0.85 | | -2.14 | | -2.31 | |
| Item 25 | | | 0.68 | | 0.47 | | 0.35 | | 0.28 | | |  | | | 0.37 | | 0.18 | | 0.93 | | 0.96 | | -0.65 | | -0.54 | |
| Item 26 | | | 0.72 | | 0.45 | | 0.26 | | 0.18 | | |  | | | 0.58 | | 0.19 | | 1.00 | | 1.02 | | 0.04 | | 0.24 | |
| Item 27 | | | 0.68 | | 0.47 | | 0.23 | | 0.15 | | |  | | | 0.37 | | 0.18 | | 1.18 | | 1.05 | | 1.57 | | 0.65 | |
| Item 28 | | | 0.49 | | 0.50 | | 0.28 | | 0.20 | | |  | | | -0.47 | | 0.17 | | 1.03 | | 1.03 | | 0.49 | | 0.63 | |
| Item 29 | | | 0.60 | | 0.49 | | 0.38 | | 0.31 | | |  | | | 0.00 | | 0.17 | | 0.99 | | 0.95 | | -0.11 | | -0.92 | |
| Item 30 | | | 0.62 | | 0.49 | | 0.36 | | 0.28 | | |  | | | 0.09 | | 0.17 | | 0.93 | | 0.97 | | -0.82 | | -0.56 | |
| Item 31 | | | 0.61 | | 0.49 | | 0.44 | | 0.37 | | |  | | | 0.06 | | 0.17 | | 0.85 | | 0.91 | | -1.78 | | -1.61 | |
| Item 32 | | | 0.41 | | 0.49 | | 0.25 | | 0.16 | | |  | | | -0.83 | | 0.17 | | 1.06 | | 1.07 | | 0.76 | | 1.16 | |
| Item 33 | | | 0.41 | | 0.49 | | 0.18 | | 0.10 | | |  | | | -0.83 | | 0.17 | | 1.18 | | 1.12 | | 2.21 | | 2.07 | |
| Item 34 | | | 0.65 | | 0.48 | | 0.38 | | 0.31 | | |  | | | 0.21 | | 0.18 | | 0.91 | | 0.95 | | -0.95 | | -0.84 | |
| Item 35 | | | 0.71 | | 0.46 | | 0.19 | | 0.11 | | |  | | | 0.51 | | 0.18 | | 1.11 | | 1.06 | | 0.87 | | 0.81 | |
| Item 36 | | | 0.56 | | 0.50 | | 0.40 | | 0.33 | | |  | | | -0.18 | | 0.17 | | 0.94 | | 0.94 | | -0.73 | | -1.20 | |
| Item 37 | | | 0.66 | | 0.48 | | 0.38 | | 0.30 | | |  | | | 0.28 | | 0.18 | | 0.93 | | 0.95 | | -0.70 | | -0.76 | |
| Item 38 | | | 0.50 | | 0.50 | | 0.33 | | 0.25 | | |  | | | -0.44 | | 0.17 | | 0.96 | | 1.00 | | -0.49 | | -0.09 | |
| Item 39 | | | 0.35 | | 0.48 | | 0.38 | | 0.30 | | |  | | | -1.14 | | 0.18 | | 0.95 | | 0.95 | | -0.52 | | -0.67 | |
| Item 40 | | | 0.63 | | 0.48 | | 0.36 | | 0.28 | | |  | | | 0.15 | | 0.17 | | 0.95 | | 0.97 | | -0.49 | | -0.53 | |

*SD* = standard deviation; without CIS = without correlated item score; beta = item easiness, i.e., the more likely a person gave an altruistic response, the higher became the score; *SE* = standard error; outfit *MSQ* = mean-square outlier-sensitive fit; infit *MSQ* = mean-square information-weighted fit; outfit and infit *MSQ* are ideally equal to one, values between 0.5 and 1.5 are called “productive for measurement”, and their corresponding *t*-value should be between -1.9 and 1.9 [1], while other sources suggest to extend these borders and to focus on the *MSQ* to reduce type I errors [2].

**References**

1. Linacre JM. What do infit and outfit, mean-square and standardized mean? Rasch Meas Trans. 2002; 16. Available from: https://www.rasch.org/rmt/rmt162f.htm. Cited 6 March 2019.

2. Smith AB, Rush R, Fallowfield LJ, Velikova G, Sharpe M. Rasch fit statistics and sample size considerations for polytomous data. BMC Med Res Methodol. 2008;8: 33. doi: 10.1186/1471-2288-8-33
