# Supplementary material for: Decision-making in everyday moral conflict situations: Development and validation of a new measure
Source: PLoS One. 2019 Apr 1;14(4):e0214747. doi: 10.1371/journal.pone.0214747 (PMC6443167; doi:10.1371/journal.pone.0214747)

**S3 Table. Inter-item correlations.**

Inter-item correlations (tetrachoric correlations) of the final 40 items of the EMCS Scale. Items 1-20 are scenarios with socially close protagonists, items 21-40 are scenarios with socially distant protagonists.


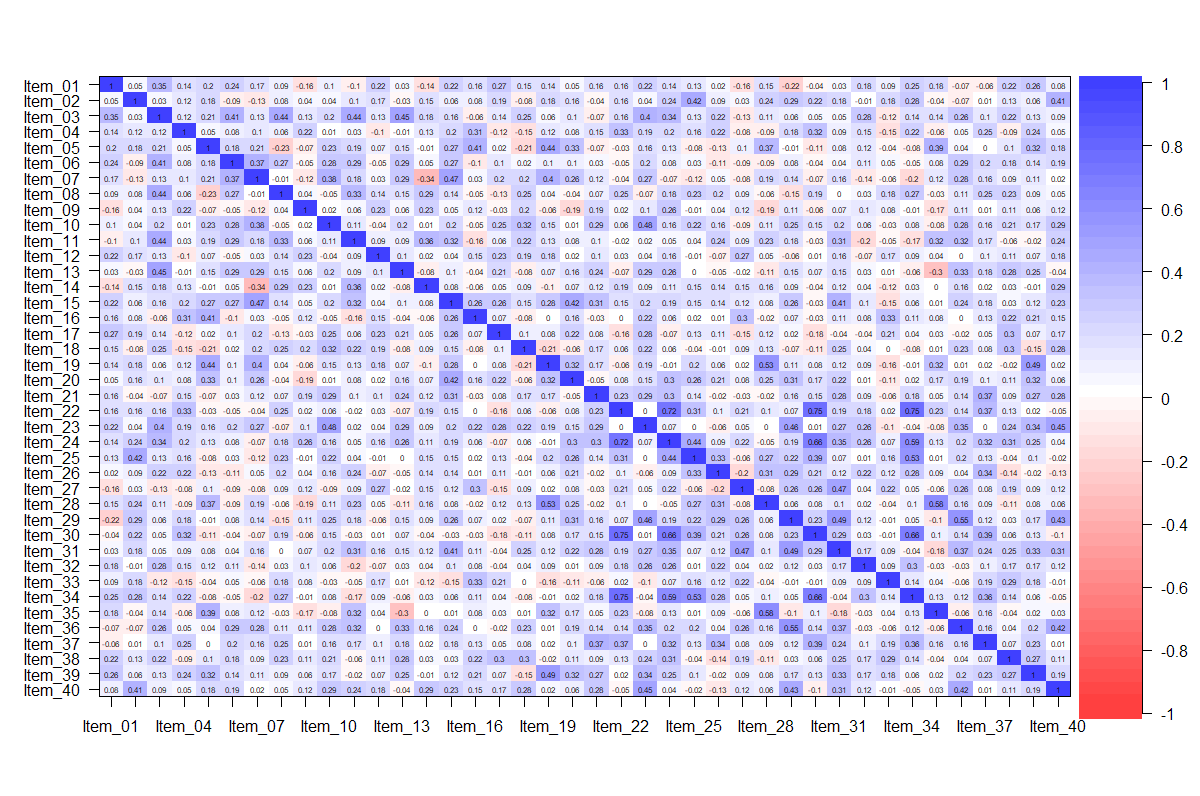

Supplement: S3 Table — shows the inter-item correlations (tetrachoric correlations) of the final 40 items of the EMCS Scale. (DOCX) [file pone.0214747.s003.docx]
